# A Review on Controlling Grain Boundary Character Distribution during Twinning-Related Grain Boundary Engineering of Face-Centered Cubic Materials

**DOI:** 10.3390/ma16134562

**Published:** 2023-06-24

**Authors:** Yu-Qing Zhang, Guo-Zheng Quan, Jiang Zhao, Yan-Ze Yu, Wei Xiong

**Affiliations:** 1 Chongqing Key Laboratory of Advanced Mold Intelligent Manufacturing, School of Material Science and Engineering, Chongqing University, Chongqing 400044, China; zhangyuqing620@sina.com (Y.-Q.Z.); 201709021061@cqu.edu.cn (J.Z.); yyz6201314@sina.com (Y.-Z.Y.); 2 Key Laboratory of Advanced Reactor Engineering and Safety of Ministry of Education, Collaborative Innovation Center of Advanced Nuclear Energy Technology, Institute of Nuclear and New Energy Technology, Tsinghua University, Beijing 100084, China

**Keywords:** grain boundary engineering, twin boundary, thermo-mechanical processing, microstructure evolution, grain boundary character distribution, properties

## Abstract

Grain boundary engineering (GBE) is considered to be an attractive approach to microstructure control, which significantly enhances the grain-boundary-related properties of face-centered cubic (FCC) metals. During the twinning-related GBE, the microstructures are characterized as abundant special twin boundaries that sufficiently disrupt the connectivity of the random boundary network. However, controlling the grain boundary character distribution (GBCD) is an extremely difficult issue, as it strongly depends on diverse processing parameters. This article provides a comprehensive review of controlling GBCD during the twinning-related GBE of FCC materials. To commence, this review elaborates on the theory of twinning-related GBE, the microscopic mechanisms used in the optimization of GBCD, and the optimization objectives of GBCD. Aiming to achieve control over the GBCD, the influence of the initial microstructure, thermo-mechanical processing (TMP) routes, and thermal deformation parameters on the twinning-related microstructures and associated evolution mechanisms are discussed thoroughly. Especially, the development of twinning-related kinetics models for predicting the evolution of twin density is highlighted. Furthermore, this review addresses the applications of twinning-related GBE in enhancing the mechanical properties and corrosion resistance of FCC materials. Finally, future prospects in terms of controlling the GBCD during twinning-related GBE are proposed. This study will contribute to optimizing the GBCD and designing GBE routes for better grain-boundary-related properties in terms of FCC materials.

## 1. Introduction

Grain boundary engineering (GBE) was developed based on the conception of “grain boundary design and control” proposed by Watanabe et al. [1] in order to strengthen the intergranular degradation resistance of materials. Until now, it has been widely used to enhance the grain-boundary-related properties in many face-centered cubic (FCC) materials with low and medium stacking fault energies (SFEs), such as austenitic stainless steels [2,3], Ag-Pd alloys [4], nickel-based alloys [5,6], and copper alloys [7]. The enhancement of properties is ascribed to the optimization of the grain boundary character distribution (GBCD), which is achieved by increasing the proportion of special twin boundaries and disrupting the connectivity of the random boundary network [8,9]. According to the coincidence site lattice (CSL) model of the interface structure, these special twin boundaries are defined as low-ΣCSL boundaries (usually 1 < Σ ≤ 29). Among the low-ΣCSL boundaries, the Σ3*^n^* (*n* = 1, 2, 3) twin boundaries account for the highest proportion. These boundaries possess lower interface energy and higher atomic ordering compared to random grain boundaries (Σ > 29), showing attractive performance in terms of ductility [6,10,11], fracture [12,13], oxidation resistance [14], corrosion resistance [15,16], sensitization [17,18], solute segregation resistance [19], and creep resistance [20]. Hence, the pursuit of a high proportion of Σ3*^n^* twin boundaries and a suitably disrupted random boundary network in microstructures has become the research focus of optimizing GBCD.

Understanding the formation mechanisms of special twin boundaries is important for GBCD optimization, which has attracted the attention of researchers. So far, the main theories for expounding the formation mechanisms of annealing twins comprise four groups: growth accident theory [21,22], grain encounter model [23,24,25], grain boundary dissociation [26,27,28], and the nucleation of twins by stacking faults or fault packets [29,30]. Although the growth accident theory has been widely accepted by many researchers, there is still no consensus on the formation mechanisms of annealing twins. In addition, some researchers also proposed microscopic models to explain the intrinsic mechanisms of optimizing GBCD induced by annealing twins, such as the Σ3 regeneration model [31], high-ΣCSL boundary decomposition model [27], the migration of incoherent Σ3*^n^* twin boundaries model [32], and special fragmentation model [3]. These models are developed to account for observed experimental phenomena, while the underlying mechanism remains controversial.

It is to be noted that during the twinning-related GBE of FCC materials, the four aforementioned microscopic models are not directly applicable for optimizing GBCD, which heavily relies on processing parameters. In order to realize GBCD control, it is imperative to elucidate the influence of processing parameters on the evolution of special twin boundaries, which has engendered sustained interest. The majority of current research employs the proportion of twin boundaries and the connectivity of random boundary networks as evaluation indicators, with extensive investigations into the relationships between these indicators and diverse processing parameters. The primary processing route for GBE is thermo-mechanical processing (TMP), which involves a series of sequential steps, including hot/cold deformation followed by an annealing treatment [33,34,35]. The initial state [33,36], deformation amount [3,37,38,39,40], annealing parameters (temperature and time) [37,41,42,43], and processing pass (single or iterative) [32,44,45,46] are the most frequently investigated factors affecting the formation of special twin boundaries. The optimization of GBCD can be achieved by adjusting these TMP parameters. Nevertheless, when it comes to specific materials employed in high-temperature applications within the aerospace, chemical, and power industries, such as nickel-based alloys, their components are commonly fashioned via thermal forming processes, such as hot forging, extrusion, and electric upsetting so as to attain exceptional mechanical properties under elevated temperatures [47,48,49,50]. The existing TMP routes for achieving GBE are not ideally suitable for these components. Souai et al. [51] investigated the dependence of annealing twin formation on processing parameters in hot deformation and demonstrated the possibility of twinning-related GBE via hot-working in a nickel-based superalloy. To some extent, it offers an opportunity to optimize the GBCD during the thermal deformation process of nickel-based alloys. Worthy of note is that during a TMP treatment or a thermal deformation process, dynamic recrystallization (DRX), dynamic recovery (DRV), grain growth (GG) mechanisms, and the formation of annealing twins exist and interact with each other [35,52,53,54,55,56]. The intricate microstructural mechanisms, which are significantly influenced by TMP treatment or thermal deformation parameters (temperature, strain rate and strain), give rise to the complex evolution process of special twin boundaries. To obtain the desired GBCD during the twinning-related GBE of FCC materials, there is a great interest in uncovering the internal evolution mechanisms of microstructures under different TMP treatment and thermal deformation parameters [53,57,58,59,60]. Furthermore, based on the growth accident theory, some researchers have directed their attention towards crafting twinning-related kinetics models that are linked to processing parameters and microstructural parameters, including grain size, stored energy, etc., to predict the evolution of special twin boundaries [21,22,61,62,63,64,65,66]. These works help to achieve GBCD control during the twinning-related GBE of FCC materials.

It is well accepted that the comprehensive properties of alloys are closely associated with microstructure characteristics, which in turn are predominantly determined via various processing parameters. Numerous studies have indicated that microstructures featuring a high proportion of special twin boundaries and the disrupted connectivity of the grain boundary network are instrumental in improving grain-boundary-related properties [2,3,5,12,13,16,67,68,69,70,71]. In this regard, it is crucial to optimize the processing parameters and untangle the complex relationships between the twinning-related microstructures and the grain-boundary-related properties. This review presents a comprehensive overview of GBCD control during the twinning-related GBE of FCC materials. In Section 2, the theory of twinning-related GBE, microscopic mechanisms for optimizing GBCD, and the optimization objectives of GBCD are expounded. In Section 3, the influence of the processing parameters on the twinning-related microstructures is discussed. Additionally, the development of twinning-related kinetics models is presented in Section 4. Finally, Section 5 delves into the application of twinning-related GBE for enhancing mechanical properties and corrosion resistance.

## 2. Twinning-Related Grain Boundary Engineering

### 2.1. Coincident Site Lattice Model

The coincidence site lattice (CSL) model was introduced by Kronberg and Wilson [72] during their investigation of secondary recrystallization in copper. It has become commonly used to describe grain boundary structure based on the misorientation angle between neighboring grains, denoted as Σ value [73]. In the CSL model, there are two lattices with the same crystal structure, each extending infinitely into space, and one crystal revolves at a certain angle *θ* around a lower exponential axis <uvw> with respect to the other crystal. Some of the points in the two crystal lattices will coincide regularly, and the new space lattice formed by the points at the overlap position is called the overlap position lattice. The numerical value of the overlap position lattice can be calculated using Equations (1) and (2) [54].

(1) $$\theta =2arctg\left[\frac{y}{x}\times {\left(u^{2}+v^{2}+w^{2}\right)}^{1/2}\right]$$

(2) $$\Sigma =x^{2}+\left(u^{2}+v^{2}+w^{2}\right)\times y^{2}$$

where *x* and *y* are integers without factors, Σ represents the ratio of CSL cell volume to lattice cell volume, which needs to be the least odd number; 1/Σ represents the coincidence location density, and the smaller the Σ value, the greater the coincidence location density.

According to the CSL model, grain boundaries can be classified into three categories: low-angle grain boundaries (LAGBs), which refer to Σ1 grain boundaries, low-ΣCSL boundaries (1 < Σ ≤ 29), also known as special twin boundaries; and high-ΣCSL boundaries (Σ > 29), referred to as random high-angle grain boundaries (HAGBs) [56,74]. Studies have indicated that low-ΣCSL boundaries, which possess relatively lower free energy compared to HAGBs, exhibit superior resistance to corrosion and segregation [75]. Table 1 lists the rotation axis <uvw> and rotation angle *θ* of low-ΣCSL boundaries. The CSL boundaries can be distinguished and characterized through the use of the electron back-scattered diffraction (EBSD) technique. There are two main standards for grain boundary measurement based on the difference in maximum deviation angle Δ*θ*_max_, namely Brandon standard (Δ*θ*_max_ = 15°Σ^−1/2^) [76] and Palumbo–Aust standard (Δ*θ*_max_ = 15°Σ^−5/6^) [77].

### 2.2. Morphology of Annealing Twin

**Figure 1:**
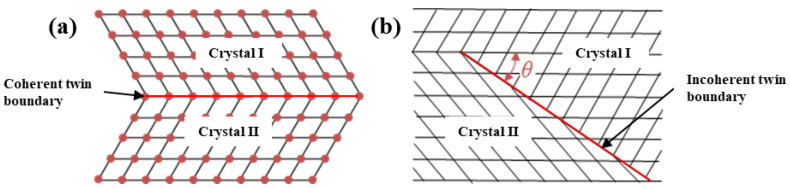
Schematics depicting coherent twin boundary (**a**) and incoherent twin boundary (**b**).

The presence of an annealing twin is critical to realizing twinning-related GBE of FCC materials. There exists an orientation relationship of <111>/60° between the annealing twin and matrix grain. That is, the annealing twin corresponds to the Σ3 twin boundary in the CSL model. According to the atomic arrangement at the interface of the adjacent crystal lattice, the annealing twin can be classified into two types [55]: coherent twin boundary and incoherent twin boundary, as schematically illustrated in Figure 1a,b. The coherent twin boundary is a twin plane on which the crystals on both sides are symmetric, forming a mirror-like symmetrical relationship. The atoms lying on the twin plane are shared by the two adjacent crystal lattices, resulting in very low and stable interfacial energy. A coherent twin boundary accounts for the highest content among the low-ΣCSL boundaries. Regarding the incoherent twin boundary, only a few atoms from the adjacent crystal lattices are shared. The interfacial energy of an incoherent twin boundary is higher than that of a coherent twin boundary but lower than that of a random grain boundary. These two types of twin boundaries often exhibit different properties.

**Figure 2:**
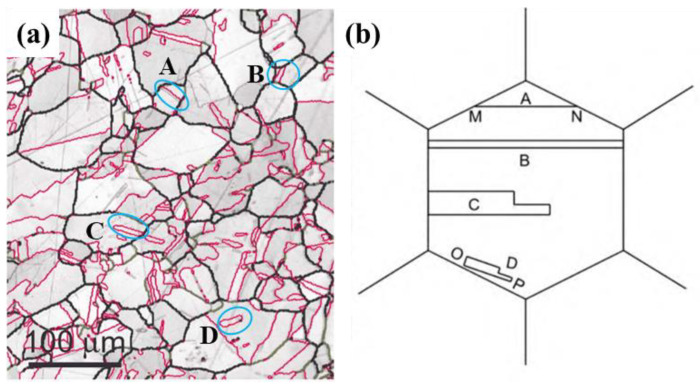
Band contrast map showing the Σ3*^n^* twin boundaries in ofe Cu (**a**) [27] and schematics of four typical morphologies of annealing twins (**b**) [29].

It should be noted that the morphologies of twin boundaries are different due to the distinct formation mechanisms of the annealing twin. The most prevalent morphology of the twin boundary, originating from or terminating at the random grain boundary, is situated within grains with a linear shape and occurs in pairs. Figure 2a exhibits the band contrast map showing Σ3*^n^* twin boundaries in oxygen-free electronic (ofe) Cu. The typical morphologies of the Σ3 twin boundary can be divided into four types, as shown in Figure 2b. Type A twins exist separately rather than in pairs. They are angular twins formed at the angular boundary of grain boundaries, and M N grain boundaries of the matrix are coherent twin boundaries. Type B twins are long strip Σ3 twin boundaries that cross the entire grain and exist in pairs. Type C twins start at a grain boundary and end inside the grain. Type D twins are internal twins occurring at trigeminal boundaries.

### 2.3. Microscopic Mechanisms for Optimizing Grain Boundary Character Distribution

The optimization of GBCD through the GBE treatment is widely acknowledged to enhance the grain-boundary-related properties of FCC materials. However, there is no agreement on the microscopic mechanisms of GBCD optimization induced by annealing twins. So far, four theories have been proposed to expound the formation mechanisms of annealing twins. The first theory is the growth accident theory [21,22], suggesting that during grain boundary migration, the coherent twin boundary is formed by the grain growth accident due to a stacking error. The second is the grain encounter model [23,24,25], which proposes that different grains initially separated encounter each other during grain growth. If these grains happen to be in twin orientation to each other, the boundary between them becomes a coherent twin boundary by reorienting itself. The third is grain boundary dissociation [26,27,28], which suggests that a general high-angle boundary can be dissociated into an incoherent twin boundary, a coherent twin boundary, and a lower energy boundary, as this reaction is interfacial energy favorable. The fourth involves the nucleation of twins due to stacking faults or fault packets [29,30]. This model believes that during the process of grain boundary migration, a twin is nucleated with its incoherent segment remaining at the grain boundary. The twin then grows by the migration of the other incoherent boundary. However, it remains unclear what drives this migration.

**Figure 3:**
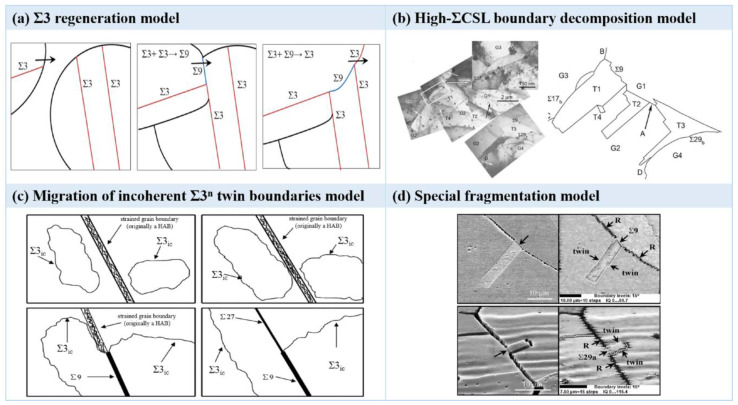
Schematic illustration of mechanisms and models for optimizing GBCD. (**a**) Σ3 regeneration model [31]; (**b**) high-ΣCSL boundary decomposition model [27]; (**c**) migration of incoherent Σ3*^n^* twin boundaries model [32]; (**d**) special fragmentation model [3].

To date, there are four models for elucidating the intrinsic mechanisms of GBCD optimization induced by annealing twins, which include the Σ3 regeneration model [31], high-ΣCSL boundary decomposition model [27], migration of incoherent Σ3*^n^* twin boundaries model [32] and special fragmentation model [3], as illustrated in Figure 3a–d. Randle et al. [31] proposed the Σ3 regeneration model, which suggests that the Σ3 twin boundary comes from the interaction of Σ3*^n^* (*n* = 1, 2, 3) twin boundaries when the proportion of the Σ3 twin boundary is relatively high. The interaction can be stated by the following rules concerning the joining or dissociation of CSL boundaries, $\Sigma 3+\Sigma 3^{n}\to \Sigma 3^{n+1}$ and $\Sigma 3^{n}+\Sigma 3^{n+1}\to \Sigma 3$. That is, the meeting of two coherent Σ3 twin boundaries will result in forming a highly mobile Σ9 twin boundary. When the Σ9 twin boundary encounters another coherent Σ3 twin boundary, an inherent Σ3 twin boundary with higher mobility is generated. Once the coherent Σ3 twin boundary meets other grain boundaries, low-ΣCSL boundaries will occur, and in doing so, the GBCD optimization of materials can be achieved. It is worth noting that in the Σ3 regeneration model, only the interaction of Σ3 twins generates incoherent Σ3 twin boundaries. The optimization of GBCD cannot be achieved solely through the presence of coherent Σ3 twin boundaries, as lower energy Σ3 twin boundaries generate relatively high-energy incoherent Σ3 twin boundaries, which violates the energy principle. Kumar et al. [27] proposed the high-ΣCSL boundary decomposition model, which holds that after undergoing proper deformation and high-temperature annealing, the Σ3 twin boundary generated via strain-induced boundary migration (SIBM) will react with high-ΣCSL boundaries to generate abundant low-ΣCSL boundaries. Wang et al. [32] proposed the migration of incoherent Σ3*^n^* twin boundaries model. It is believed that a large number of incoherent Σ3 twin boundaries will form during the recrystallization–annealing process, and these boundaries will react with other special twin boundaries or RHAGBs to produce special twin boundaries. The resultant grain cluster and trigeminal grain boundaries can effectively interrupt the connectivity of the random boundary network. According to the special fragmentation model proposed by Shimada et al. [3], the annealing twins introduce low-energy special twin boundaries into high-energy random grain boundaries through divergence so as to optimize GBCD. It should be noted that these models are supported by experimental results in their works. The different formation mechanisms of annealing twins may act simultaneously in the same material, and what factors dominate the mechanism need to be further studied.

### 2.4. Optimization Objectives of Grain Boundary Character Distribution

The optimization of the GBCD in microstructures is manipulated with the objective of strengthening the grain-boundary-related properties. This portion provides a description of how to optimize GBCD during the twinning-related GBE of FCC materials. The optimization objectives of GBCD, including the proportion of special twin boundaries and the connectivity of the random boundary networks, are summarized.

#### 2.4.1. Proportion of Special Twin Boundaries

The proportion of special twin boundaries is a commonly used indicator to evaluate the optimization effect of GBCD. Specifically, the fraction and density of special twin boundaries that are employed to quantify this proportion [42,66]. The former is defined as the ratio of the total length of all Σ3*^n^* twin boundaries to the total length of all high-angle grain boundaries, and the latter corresponds to the total length of Σ3*^n^* twin boundaries per unit area. As reported by numerous researchers [68,79,80,81,82], increasing the proportion of special twin boundaries by adjusting the processing parameters is an effective method for improving grain-boundary-related properties in many FCC materials.

#### 2.4.2. Connectivity of Random Boundary Network

The point worth mentioning about GBCD optimization is that aside from the proportion of special twin boundaries, whether these low-ΣCSL boundaries can effectively interrupt the connectivity of random grain boundaries should be considered. Lehockey et al. [83] demonstrated that the intergranular failure resistance of a material can only be improved when the connectivity of the random boundary network is interrupted by low-ΣCSL grain boundaries. Currently, the primary methods for characterizing the random boundary network connectivity include the following aspects: triple junction character distribution, grain cluster, percolation theory and fractal analysis.

(a) Triple junction character distribution

**Figure 4:**
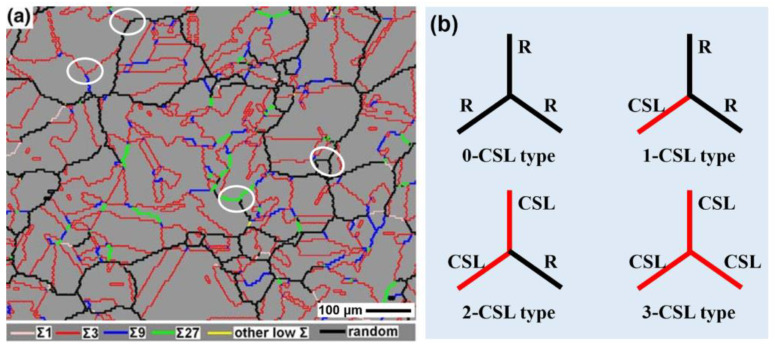
GBCD map in fully recrystallized sample of nickel-based alloy 690 (**a**) [87] and schematic diagram of different triple junction types (**b**).

According to the number of low-ΣCSL boundaries in the triple junction (TJ), there are four distinct types: three random boundaries (R-R-R or 0-CSL), one special and two random boundaries (S-R-R or 1-CSL), two special boundaries and one random boundary (S-S-R or 2-CSL), and three special boundaries (S-S-S or 3-CSL) [84,85,86]. To clarify these triple junction types more clearly, an example of grain boundary distribution in a fully recrystallized sample of nickel-based alloy 690 is given in Figure 4a. Numerous Σ3*^n^* twin boundaries are evidently present, forming many triple junction types, as indicated by white circles. These different types are schematically illustrated in Figure 4b, with black lines representing random grain boundaries and red lines denoting the low-ΣCSL boundaries.

(b) Grain cluster

**Figure 5:**
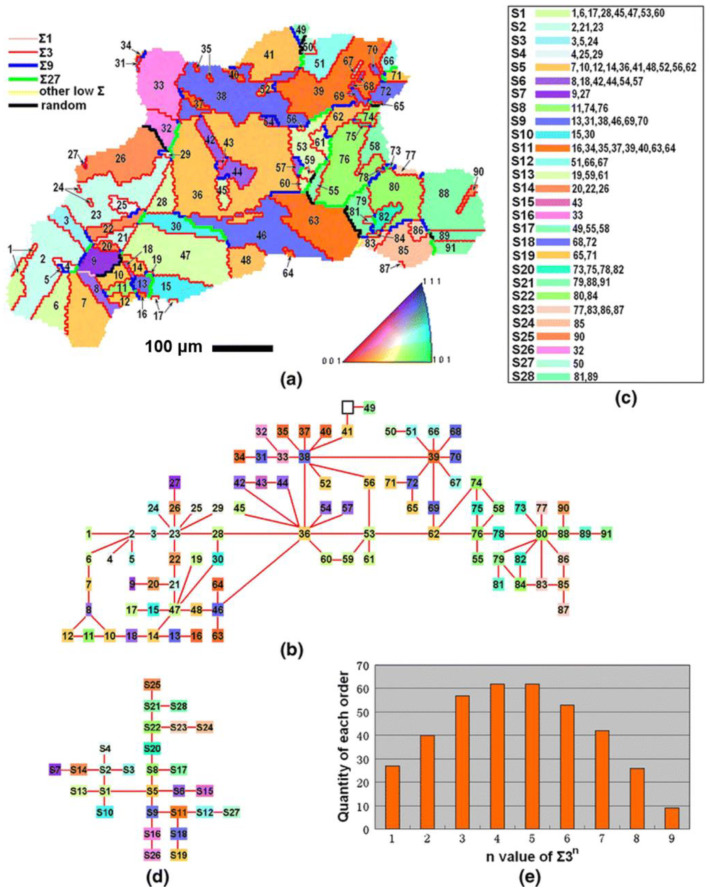
Twin chain analysis of large grain cluster, as shown in Figure 4a [87]: (**a**) Grain cluster containing 91 grains; (**b**) twin chain of those 91 grains; (**c**) these 91 grains were categorized into 28 orientations; (**d**) twin chain of these 28 orientations; (**e**) quantity of each order twin relationships.

A grain cluster, also referred to as the twin-related domain (TRD), is represented by a region composed of grains exhibiting a Σ3*^n^* orientation relationship with each other. The inner structure of a grain cluster is composed of Σ3 twin boundaries and their geometrically related twin boundaries (Σ9, Σ27), while the outer grain is composed of random HAGBs. It has been reported that a grain cluster is formed due to multiple twinning, i.e., the iterative process of twinning operations starting from a single nucleus during recrystallization [16,36,87,88,89]. Generally, a tree-shape twin chain is reconstructed to explore the behaviors of multiple twinning. Xia et al. [87] investigated and statistically analyzed the grain cluster microstructure of Alloy 690. The twin chain of a large grain cluster containing 91 grains, shown in Figure 4a, was constructed, as exhibited in Figure 5. It is observed that all grains exhibit Σ3*^n^* mutual misorientation regardless of whether they are adjacent, and twin relationships up to the ninth generation (Σ3^9^) exist in this large grain cluster. Such a grain cluster plays a crucial role in disrupting the connectivity of the random boundary network, which is beneficial to improving the intergranular corrosion resistance of materials [16,79,82,90]. Consequently, the formation of the grain cluster is an important feature of GBCD optimization.

(c) Percolation theory

Percolation theory is a stochastic geometric probability model that can be utilized to quantitatively characterize the connectivity of random boundary networks and predict the probability of material failure [91]. Some researchers commonly employ percolation models to determine the probability of intergranular crack arrest at triple junctions and subsequently evaluate the impact of optimized GBCD via the GBE treatment on a material’s resistance to intergranular cracks [40,86,92,93,94]. Summarizing these works, three percolation models are generally used to estimate the probability of intergranular crack arrest at triple junctions.

Kumar et al. [95] proposed a judging condition for effectively preventing grain boundary failure with triple junctions, as shown in the use of Equation (3).

(3) $$P_{K}=f_{2-CSL}/(1-f_{3CSL})\geq 0.35$$

where $f_{2-CSL}$ is the fraction of the 2-CSL triple junction type that are thus able to arrest intergranular cracks. $\left(1-f_{3CSL}\right)$ is the fraction of the triple junctions that are active unit entities in the microstructure. When the above formula condition is satisfied, the connectivity of the random boundary network can be effectively disrupted, and the propagation path of cracks can be broken.

In Kumar’s model, crack arrest probability, $P_{K}$, is calculated from the type of triple junctions. Considering the contribution of the fraction of special twin boundaries, Palumbo et al. [96] derived a formula to calculate the probability of intergranular crack arrest at a given triple junction $P_{p}$, as shown in the use of Equation (4).

(4) $$P_{p}=f_{\mathrm{sp}}^{2}+2\left[f_{0}f_{\mathrm{sp}}(1-f_{\mathrm{sp}})\right]$$

where $f_{\mathrm{sp}}$ is the fraction of special twin boundaries that are not susceptible to cracking, and $f_{0}$ is the fraction of grain boundaries that are unfavorably oriented to the stress axis.

Marrow et al. [97] observed that cracks arrest at the 1-CSL type and 2-CSL type triple junctions, and proposed another model for calculating the probability of intergranular crack arrest at triple junctions $P_{M}$, as shown in the use of Equation (5).

(5) $$P_{M}=\frac{f_{a}f_{2-\mathrm{CSL}}+f_{b}f_{1-\mathrm{CSL}}}{1-f_{3-\mathrm{CSL}}}$$

where $f_{a}$ and $f_{b}$ are geometrical factors that are used to account for unfavorably oriented sensitized boundaries at the triple junction, usually tanking as 1 and 0.5, respectively.

(d) Fractal analysis

**Figure 6:**
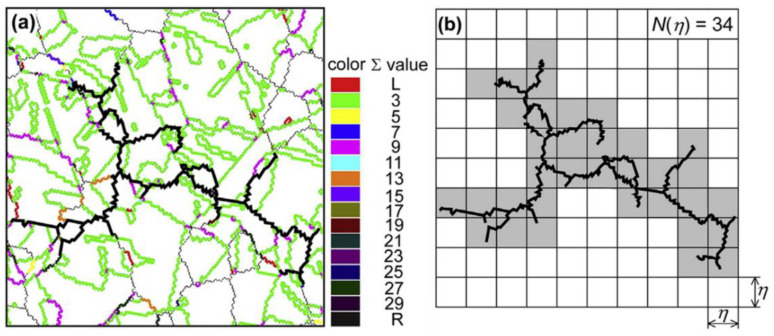
An example of maximum random boundary network connectivity (**a**) and its fractal dimension calculated through the box-counting method (**b**) [2].

The fractal analysis of random boundary network connectivity is evaluated based on the box-counting method [2,13,18,39]. The fractal dimension of the maximum random boundary network connectivity related to the propagation path of intergranular corrosion is estimated based on the grain clusters in the microstructures. Figure 6 provides an example of the maximum random boundary network connectivity, as represented by the square boxes with a size of η. The total number of shaded square boxes required for complete coverage of the maximum random boundary network connectivity amounts to N(η)=34. Due to the self-similar nature of the maximum random boundary network connectivity, the relationship between N(η) and η can be described as presented in Equation (6). The fractal dimension of the maximum random boundary network connectivity *D_R_* can be calculated by the slope of the linear relationship between  logN(η) and log(η), as expressed in Equation (7).

(6) $$N(\eta )\propto \eta^{-D_{R}}$$

(7) $$D_{R}=\frac{-\log N(\eta )}{\log(\eta )}$$

## 3. Microstructure Control for Optimizing Grain Boundary Character Distribution

**Figure 7:**
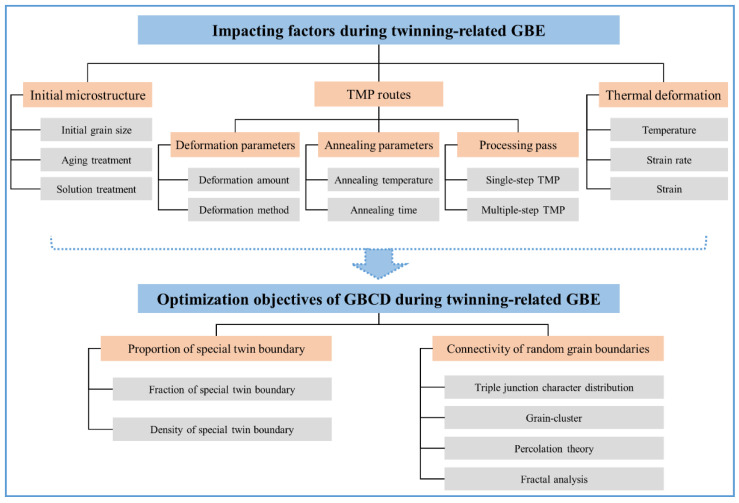
Summary of impacting factors and optimization objectives of GBCD during twinning-related GBE.

During the twinning-related GBE of FCC materials, GBCD optimization signifies the improvement of special twin boundaries, which is concretely shown in increasing the proportion of special twin boundaries and disrupting the connectivity of the random boundary network [8,9,55]. Therefore, a thorough investigation of the influence of the processing parameters on microstructure evolution and related mechanisms is vital for achieving GBCD control. This is more significant in enhancing the grain-boundary-related properties of FCC materials. Figure 7 summarizes the impacting factors and optimization objectives of GBCD during twinning-related GBE. Herein, the impacting factors are classified into three aspects: initial microstructure, thermo-mechanical processing routes, and thermal deformation. In twinning-related microstructures, the optimization objectives of GBCD include the proportion of special twin boundaries and the connectivity of random grain boundaries. In the following sections, the influence of the processing parameters on the evolution of twinning-related microstructures is highlighted and thoroughly discussed.

### 3.1. Influence of Initial Microstructure

The initial state of a specimen is an important factor that affects GBCD during the twinning-related GBE. Some studies have analyzed the influence of the initial microstructures, such as the initial grain size, carbide precipitation, etc., on the twining-related microstructures during material TMP processes. The study conducted by Feng et al. [33] suggested that initial grain size has an important influence on the grain growth behaviors and GBCD of austenitic 304 stainless steel. Although a smaller initial grain size (10.6 μm) increases the proportion of low-ΣCSL boundaries, it also results in the random grain boundaries presenting with a continuous network. On the contrary, a larger initial grain size (34.9 μm) not only increases the proportion of low-ΣCSL boundaries but also interrupts the connectivity of the random boundary network. However, there is no significant effect on the GBCD when the initial grain size is fairly large at 48.7 μm, while abnormal grain growth will still occur. Liu et al. [36] studied the effect of different initial grain sizes on the twinning-related microstructures of Alloy 690. The results revealed that the initial grain size has a combined impact on the proportion of low-ΣCSL boundaries, grain cluster size, and final grain size. Twin density increases with the decrease in the initial grain size, while it hinders the formation of large grain clusters, which is not conducive to improving the proportion of low-ΣCSL boundaries. Li et al. [98] conducted a comparison of the grain boundary network in nickel-based Alloy 690 under solution-annealed and aging-treated conditions. The results indicated that the pre-existing carbides in the aging-treated specimens impede recrystallization by obstructing grain boundary migration, which requires a longer annealing time or higher annealing temperature to increase the proportions of low-ΣCSL boundaries. The results of Cao et al. [99] also showed that the initial grain size and primary carbide distribution exert a significant impact on recrystallization, thus determining the grain cluster formation and GBCD of Hastelloy N alloy. Liu et al. [100] conducted heat treatment experiments on Fe-Mn-Al-C-Cr steel using specimens with different initial fractions of special boundaries and found that the pre-existing special boundaries can inhibit grain growth during annealing. Summarizing these studies, it is suggested that selecting an appropriate initial grain size or performing an appropriate pre-treatment contributes to optimizing GBCD in material TMP treatments.

### 3.2. Influence of Thermo-Mechanical Processing Routes

TMP treatment is a complex process that involves two critical processing stages: prior cold/hot deformation and subsequent annealing. The TMP routes for achieving GBE can be categorized into two strategies, namely strain annealing and strain recrystallization [7,34]. It should be pointed out that these strategies do not, in fact, involve separate mechanisms. GBE can be achieved in either strategy, provided that grain boundary migration occurs and is accompanied by the formation of twins. Additionally, there must be an opportunity for Σ3 twin boundaries and their variants to interact and subsequently interrupt the random boundary network [55]. In TMP routes, the amount of deformation, annealing parameters (annealing temperature and annealing time), and processing pass (single-step or multiple-step TMP) are critical processing parameters that significantly impact the formation of low-ΣCSL boundaries. To optimize the GBCD through the use of a TMP treatment, extensive investigations have been conducted to explore the influence of the processing parameters on the proportion of low-ΣCSL boundaries, the connectivity of the random boundary network, and the internal evolution mechanisms of the microstructures during the twinning-related GBE of FCC materials.

**Figure 8:**
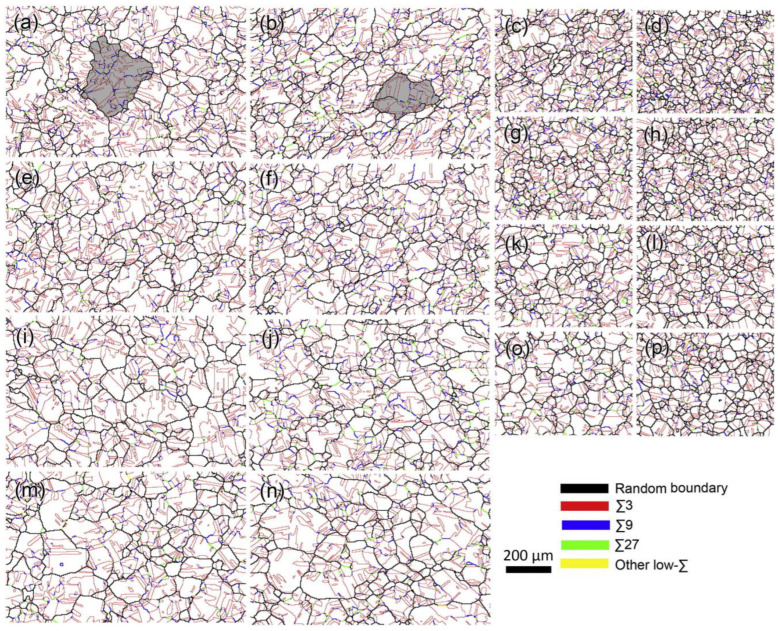
GBCD maps of nickel-based alloy 825 after cold deformation at 3% (**a**,**e**,**i**,**m**), 5% (**b**,**f**,**j**,**n**), 7% (**c**,**g**,**k**,**o**), and 10% (**d**,**h**,**l**,**p**) and heat treated at 1050 °C (**a**–**d**), 1075 °C (**e**–**h**), 1100 °C (**i**–**l**), and 1125 °C (**m**–**p**) for 10 min [58].

**Figure 9:**
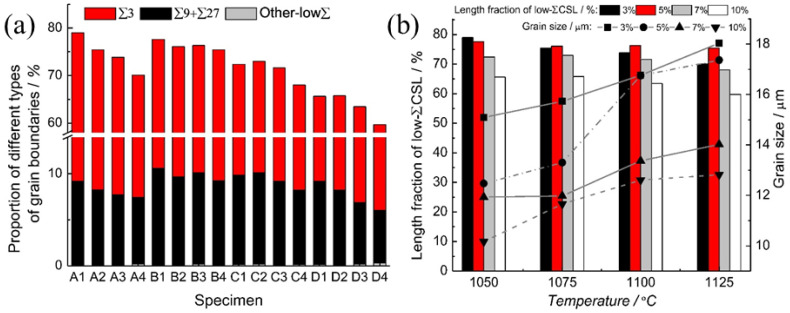
Proportion of different types of grain boundaries in specimens of nickel-based alloy 825 **(a)** and the effect of deformation amount and annealing temperature on the length fraction of low-ΣCSL boundaries and average grain size **(b)** [58]. (A1: 3% + 1050 °C/10min; A2: 3% + 1075 °C/10 min; A3: 3% + 1100 °C/10 min A4: 3% + 1125 °C/10 min; B1: 5% + 1050 °C/10 min; B2: 5% + 1075 °C/10 min; B3: 5% + 1100 °C/10 min; B4: 5% + 1125 °C/10 min).

Bai et al. [58] analyzed the influence of the amount of deformation and annealing temperature on the proportion of low-ΣCSL boundaries during the TMP treatment of nickel-based alloy 825 tubes. The results showed that the proportion of Σ3*^n^* twin boundaries increases to more than 75% after undergoing 5% cold drawing and subsequent annealing at 1050 °C for 10 min. Figure 8 shows the GBCD maps of nickel-based alloy 825 after TMP treatments with different deformation amounts of 3%~10% and annealing temperature of 1050~1125 °C. According to the microstructure analysis, abundant Σ3*^n^* twin boundaries are observed as being interconnected with each other, forming many triple junctions of Σ3*^n^* twin boundaries. The Σ3*^n^* twin boundaries are surrounded by random HAGBs, and the size of the grain clusters (indicated by the gray background in Figure 8) varies significantly under different amounts of deformation. Figure 9 shows the length fraction of low-ΣCSL boundaries and the average grain size under different TMP routes, whereas Figure 9a presents a detailed breakdown of Σ3, Σ9 + Σ27, and other low-ΣCSL boundaries proportions. The amount of deformation and annealing temperature are demonstrated as exerting a remarkable influence on the proportion of low-ΣCSL boundaries and the average grain size. Among all of the specimens, specimen A1 exhibits the highest proportion of low-ΣCSL boundaries after undergoing TMP routes with 3% cold drawing and 1125 °C annealing treatment for 10 min. Under deformation amounts of 3%, 7% and 10%, with the increase in the annealing temperature, the fraction of low-ΣCSL boundaries decreases as the grain size increases. Under the deformation amount of 5%, there is no obvious change in the fraction of low-ΣCSL boundaries with increasing annealing temperature, while the average grain size increases.

**Figure 10:**
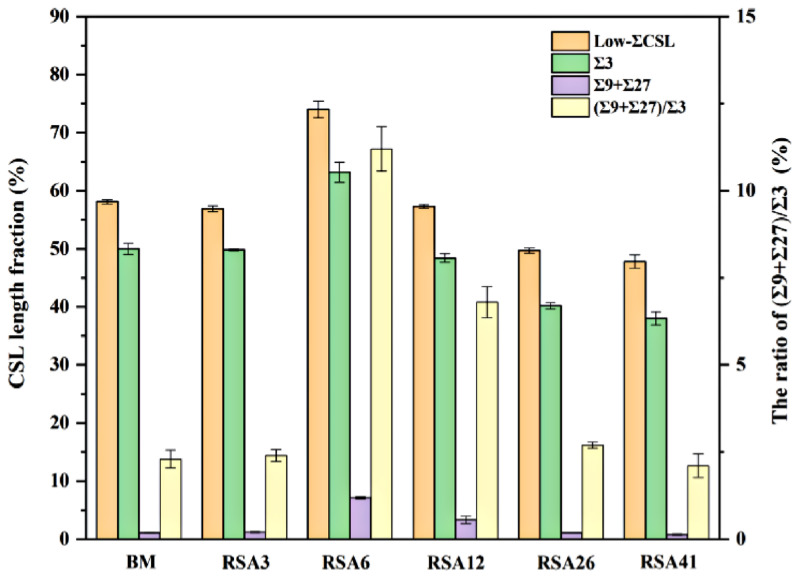
Statistic results of GBCD under different TMP routes of austenitic 304 stainless steel [90]. (BM: Initial state after pre-treatment; RSA3, RSA6, RSA12, RSA26 and RSA41 represent the BM specimens that underwent rotary swaging to different true strains of 0.03, 0.06, 0.12, 0.26, and 0.41 and then annealed at 1050 °C for 5 min).

Feng et al. [90] employed TMP routes consisting of rotary swaging deformation and an annealing treatment to optimize the GBCD of austenitic 304 stainless steel. The indicator of the (Σ9 + Σ27)/Σ3 ratio was introduced to clarify the mechanisms underlying the increase in the Σ3 twin boundaries fraction, including the new twinning formation mechanism and the Σ3 regeneration mechanism. This indicator has also been utilized to characterize the connectivity of the random boundary network in twinning-related microstructures. A higher ratio indicates the dominant role of the Σ3 regeneration mechanism. Such circumstances result in convoluted morphologies of the Σ3 twin boundaries and the potential disruption of random boundary network connectivity due to the interaction among Σ3*^n^* twin boundaries. A lower ratio represents the predominant role of the new twinning formation mechanism. The microstructures are characterized by straight and long pairs in the Σ3 twin boundaries that typically occur within grains and do not effectively interrupt the random boundary network. It should be mentioned that the increased fraction of Σ3 twin boundaries is a result of the simultaneous action of these two mechanisms, with one mechanism being dominant. Figure 10 exhibits the statistical results of GBCD optimization under different TMP routes. The findings indicate that the most effective TMP route involves rotary swaging deformation, with a true strain of 0.06, followed by an annealing treatment at 1050 °C for 5 min, resulting in a maximum fraction of low-ΣCSL boundaries (74.0%). Meanwhile, the (Σ9 + Σ27)/Σ3 ratio also reaches its largest value of 11.2%, which signifies that the Σ3 regeneration mechanism plays a dominant role.

**Figure 11:**
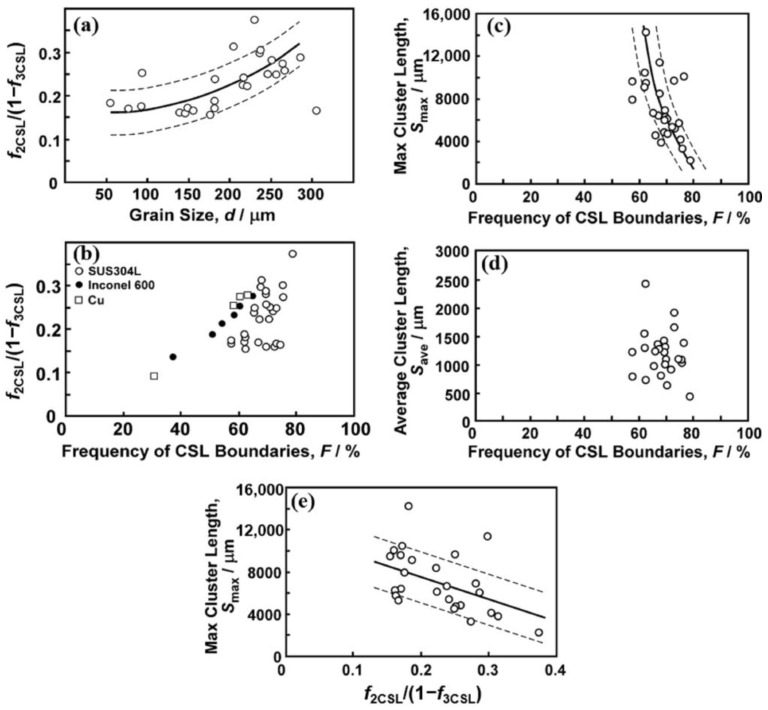
Relationships of random boundary network connectivity with grain size and GBCD [86]. (**a**) Frequency of resistant triple junction vs. grain size; (**b**) frequency of resistant triple junction vs. frequency of CSL boundaries; (**c**) maximum cluster length vs. frequency of CSL boundaries; (**d**) average cluster length vs. frequency of CSL boundaries; (**e**) maximum cluster length vs. frequency of resistant triple junction.

Tsurekawa et al. [86] focused on the relationship between random boundary network connectivity and GBCD in 304L austenitic stainless steel. In their study, the connectivity of the random boundary network was assessed based on the triple junction character distribution and the length of the grain cluster. The frequency of resistant triple junctions was determined using the method proposed by Kumar et al. [95]. Figure 11 shows the relationships of random boundary network connectivity with grain size and GBCD. It can be observed from Figure 11a,b that the frequency of resistant triple junctions increases with the increase in grain size and the frequency of CSL boundaries. It is suggested that under higher grain boundary migration, more twin boundaries are formed, and their interactions with random grain boundaries usually result in the formation of segments of CSL boundaries, which ultimately increases the frequency of resistant triple junctions. Figure 11c,d shows that the maximum cluster length decreases drastically with the increasing frequency of CSL boundaries, while the average cluster length remains relatively unaffected. Figure 11e demonstrates that despite considerable data scatter, an increase in the frequency of resistant triple junctions is likely to result in a decrease in the maximum cluster length.

With the aim of increasing the proportion of low-ΣCSL boundaries and disrupting the connectivity of the random boundary network, numerous efforts have been devoted to optimizing the processing parameters used in a single-step TMP treatment. Michiuchi et al. [101] optimized a single-step TMP treatment for austenitic 316 stainless steel and obtained a high frequency of low-ΣCSL boundaries under a pre-strain of 3% and a subsequent annealing treatment at 967 °C for 72 h. Xia et al. [87,102] investigated the influence of the strain and annealing processes on the distribution of Σ3 twin boundaries in Alloy 690. It is found that the microstructures formed under a small strain of 5% and a high temperature of 1100 °C are characterized by grain clusters that consist of triple junctions of Σ3*^n^* twin boundaries. Studies conducted by Guyot et al. [103] on Ni-200 alloy and Shimada et al. [3] on 304 stainless steel suggested that low-level strain combined with a short-time and high-temperature annealing treatment or long-time and low-temperature annealing treatment contributes to increasing the fraction of low-ΣCSL boundaries. Lee et al. [43] studied the long-term annealing behaviors of commercially pure nickel with low-level deformation under moderate annealing temperatures of 700 °C and 800 °C for 168 h. The increased special twin boundaries result in disrupting the connectivity of the random boundary network. Qian et al. [104] characterized the GBCD of a wrought Ni3Al-based alloy that underwent long-term annealing at 1180 °C for durations ranging from 10 to 40 h. The results indicated that twinning and detwinning occur and evolve competitively during the annealing process, with a reduction in the frequency of Σ3 twin boundaries observed as annealing time increases.

**Figure 12:**
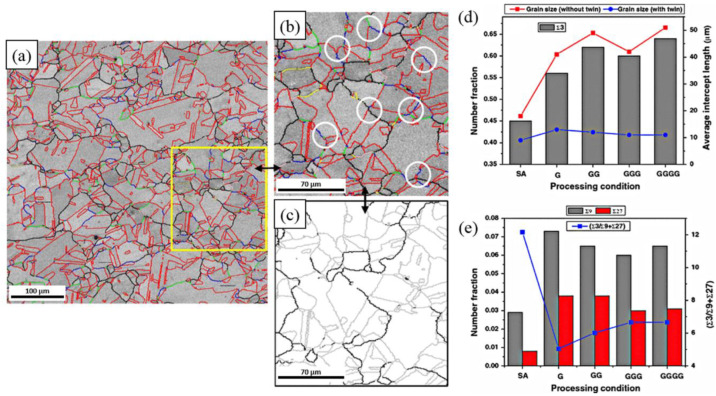
Results of GBCD under different multiple-step TMP treatments [45]. (**a**) GBCD map under the condition of GG; (**b**) multiple twinning; (**c**) reconstructed grain boundary map; (**d**) frequency distributions of Σ3 twin boundaries; (**e**) frequency distributions of Σ9 and Σ27 twin boundaries. (SA: Initial state after pre-treatment; G, GG, GGG, and GGGG represent the SA specimens subjected to 1–4 iterations TMP treatment under the conditions of 10% deformation and 1273 K/0.5 h annealing).

Additionally, certain researchers have also directed their attention toward analyzing the influence of processing passes during TMP treatment on the GBCD of FCC materials. For instance, Wang et al. [32] noted that the GBCD of Pb-Ca-Sn-Al alloy is significantly affected by TMP iterations. The connectivity of random grain boundaries is sufficiently interrupted by special twin boundaries, resulting in an increase in the fraction of low-ΣCSL boundaries to 80% after three iterations of TMP. Multiple-step TMP treatment has been shown to optimize the GBCD in various materials, such as 304L austenitic stainless steel, as by Pradhan et al. [40], Pb–0.09Ca–1.8Sn alloy, as by Lee et al. [105], ofe-Cu by Kumar et al. [27], 60Cu–40Zn two-phase alloy by Lee et al. [106], etc. It should be noted that the number of iterations has a significant but limited impact on increasing the proportion of special twin boundaries, and more iterations do not necessarily lead to a better optimization effect. Mandal et al. [45] discussed the effect of one-step and multiple-step (up to 4 cycles) TMP treatments on the promotion of Σ3*^n^* twin boundaries in Ti-modified austenitic stainless steel. Figure 12 demonstrates the optimization results of GBCD during the multiple-step TMP treatment of Ti-modified austenitic stainless steel. The results show that under identical TMP routes involving 10% deformation followed by annealing at 1273 K for 0.5 h, the fraction of Σ3 twin boundaries increases after two and four iterations of TMP, while a perceptible drop is observed after three iterations.

**Figure 13:**
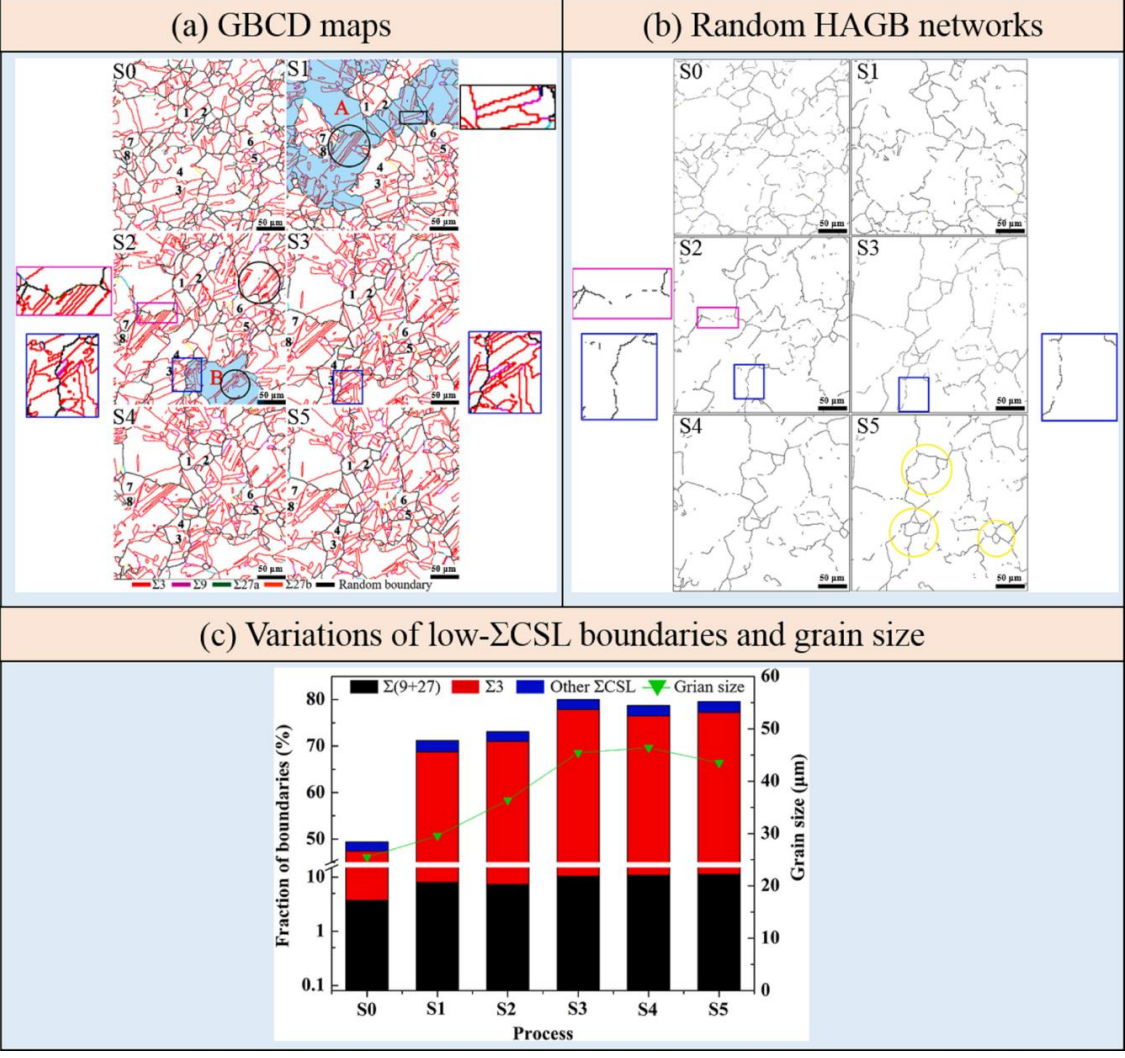
GBCD results for the 10% compression specimens annealing at 500 °C under sequential steps of S0 to S5 [59]. (**a**) GBCD maps; (**b**) random HAGB networks; (**c**) variations of low-ΣCSL boundaries and grain size.

Although great achievements in relation to the TMP treatment have been made in terms of the GBCD optimization of FCC materials with low and medium SFE, further elucidation is required to fully understand the relationship between GBCD and the mechanisms of microstructural evolution. It is still a contentious issue whether recrystallization, grain growth, or strain-induced boundary migration (SIBM) has a more efficient effect in terms of achieving the optimization of GBCD. Kumar et al. [27] utilized TEM and EBSD methods to characterize the microstructures during the GBE of Inconel 600 alloy. The results suggested that SIBM is the operative mechanism for strain-recrystallization TMP since no sign of the nucleation of new strain-free grains is found. Tokita et al. [60] successfully observed the details of the development of grain clusters and the disconnection of random grain boundaries during the single-step TMP treatment of austenitic 304 stainless steel using in situ EBSD and indicated that the growth of grain clusters was considered to be driven by strain-induced grain growth. Yang et al. [59] adopted the quasi in situ heating EBSD method to reveal the mechanisms of how SIBM optimizes the GBCD during the low-strain TMP treatment of pure copper. It is found that SIBM is more effective in achieving GBCD optimization compared to recrystallization. The primary procedure of SIBM involves the formation of numerous new Σ3 twin boundaries to increase the fraction of special twin boundaries and the introduction of low-energy segments to interrupt the connectivity of the random boundary network. Figure 13 displays the microstructure evolution of the 10% compressed specimens annealed at 500 °C under sequential steps. As indicated by black circles in Figure 13a, Σ3 twin boundaries with dense, straight and parallel morphologies are formed, typical characteristics of newly formed twins. In Figure 13b, the random boundary networks are gradually interrupted, resulting in an increased fraction of Σ3 twin boundaries and the formation of twin-related domains. Under the comprehensive effects of the interaction of Σ3*^n^* twin boundaries and the formation of a new twinning mechanism, the fractions of Σ3 and (Σ9 + Σ29) twin boundaries significantly increase from step S0 to step S3, while they remain basically unchanged under steps S4 and S5 (Figure 13c) due to insufficient stored energy to support the further progress of SIBM.

Table 2 summarizes the optimization results of GBCD during the TMP treatment of nickel-based alloys, austenitic stainless steels, Pb-alloys, and copper alloys. These works mainly focused on achieving the twinning-related GBE of materials via the optimization of the processing parameters of the TMP routes. Analyzing these investigations, it can be concluded that during the TMP routes, the determination of the annealing parameters is largely dependent on the level of pre-strain. In general, a low-level amount of deformation requires either a short-time and high-temperature annealing treatment or a long-time and low-temperature annealing treatment. A medium amount of deformation is applicable to a high-temperature and short-time annealing treatment. The specific TMP parameters should be coordinated carefully to achieve the GBCD optimization of FCC materials.

### 3.3. Influence of Thermal Deformation Parameters

For certain materials used in high-temperature applications, such as nickel-based alloys, their components are typically produced through the use of a thermal forming process to achieve exceptional mechanical properties at elevated temperatures. To this end, numerous studies have investigated the influence of deformation parameters on the evolution of annealing twins and clarified the mechanisms of microstructural evolution during thermal deformation processes. This section mainly focuses on optimizing GBCD during the thermal deformation of FCC materials, with a particular emphasis on nickel-based alloys.

**Figure 14:**
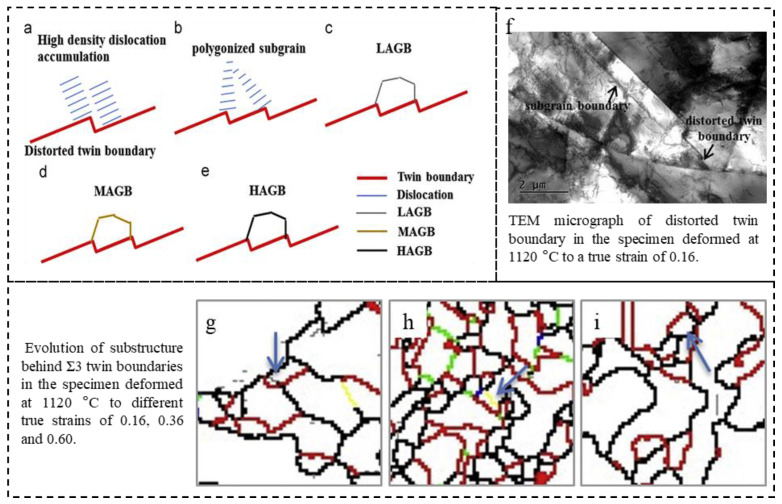
Schematic diagram of DRX nucleation and growth at the twin steps [116]. (**a**) High density dislocation accumulation; (**b**) polygonized sub-grain; (**c**–**e**) sub-grains evolve into LAGBs, MAGB, and HAGB; (**f**) TEM micrograph of distorted twin boundary in the specimen deformed at 1120 °C to a true strain of 0.16; (**g**–**i**) evolution of substructure behind Σ3 twin boundaries in the specimen deformed at 1120 °C to different true strains of 0.16, 0.36 and 0.60.

During a thermal deformation process, a variety of complex microstructural mechanisms occur, including DRX, DRV, grain growth, and twin evolution. Among these mechanisms, DRX is the dominant softening mechanism that can effectively reduce the hot-working load and form new strain-free grains with superior mechanical properties in the formed components [111,112]. It is worth noting that the occurrence of DRX is often accompanied by the formation of low-ΣCSL boundaries in FCC materials with low or medium SFE. In return, the formation of special twin boundaries also plays a crucial role in the nucleation and development of DRX grains. That is to say, annealing twins not only result from but also participate in the mechanisms of microstructural evolution [113,114,115]. Research has shown that the formation of twins accelerates the bulging of the original grain boundaries and the separation of bulged grain boundaries [116]. Wang et al. [117] revealed that Ʃ3 twin boundaries are generated at DRX grain boundaries. The formation of Ʃ3 twin boundaries during continuous dynamic recrystallization (CDRX) alters grain orientation and suppresses CDRX nucleation, whereas, in discontinuous dynamic recrystallization (DDRX), they provide additional nucleation sites and enhance the nucleation rate of DRX grains. Jiang et al. [116] analyzed the evolution of twins and substructures as well as their contribution to DRX during the low-strain-rate hot compression processing of 617B alloy. The study revealed that the twin steps formed via twin slipping provide preferential nucleation sites for DRX grains, thus facilitating the occurrence of DRX. Figure 14 shows a schematic diagram of DRX nucleation and growth under twin steps, which can be described as follows: during continuous deformation, high-density dislocations tend to accumulate at the twin step (Figure 14a) and subsequently evolve into polygonized sub-grains (Figure 14b). These sub-grains then evolve into LAGBs (Figure 14c), MAGB (Figure 14d), and HAGB (Figure 14e). The TEM micrograph depicted in Figure 14f reveals the existence of a distorted twin boundary and sub-grain boundary within the specimen that underwent deformation to a true strain of 0.16. Additionally, Figure 14g–i showcases the microstructures of specimens deformed to different true strains of 0.16, 0.36, and 0.60, which further validate the evolution of LAGB, MAGB, and HAGBs.

**Figure 15:**
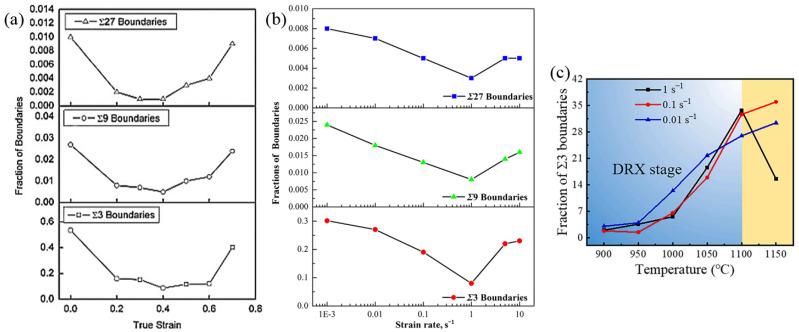
Evolution of Σ3*^n^* twin boundaries fractions with deformation parameters. (**a**) strain [118]; (**b**) strain rate [119]; (**c**) temperature and strain rate [117].

Numerous works have explored the deformation parameters that influence the evolution of annealing twins. The critical parameters discussed in this work include the thermal deformation temperature, strain rate, and strain. Figure 15a presents the variations in Σ3, Σ9, and Σ27 twin boundaries under different true strains of 800H alloy isothermally compressed under the temperature of 1150 °C and a strain rate of 1 s^−1^, as reported by Cao et al. [118]. It is found that during a compression process, the fraction of Σ3*^n^* twin boundaries initially decreases and gradually increases with increasing true strain. Studies conducted by Cao et al. [118] and Jiang et al. [116] revealed that the decreasing tendency is attributed to the deviation of pre-existing twin boundaries from their allowable limit (less than 8.71 from the reference misorientation of 60° about the <111> axis) resulting from imposed strain. Meanwhile, the twin boundaries in the initial microstructures evolve into considerable mobile grain boundaries within the deformed grains, reducing the fractions of Σ3*^n^* twin boundaries. As the strain increases continuously, there is a corresponding increase in the fractions of Σ3*^n^* twin boundaries. This can be attributed to the simultaneous generation of new Σ3 twin boundaries during the development of DRX grains. Zhang et al. [119] analyzed the influence of different strain rates on the microstructure evolution of a nickel-based alloy by means of hot compression tests. Figure 15b exhibits the evolution of the fraction of Σ3*^n^* twin boundaries under different strain rates. The results reveal that at the intermediate strain rate of 1 s^−1^, the fractions of Σ3, Σ9, and Σ27 twin boundaries are noticeably lower than those observed at lower and higher strain rates. This phenomenon can be attributed to the following reasons. At higher strain rates, higher stored energy provides a larger driving force for grain boundary migration, thereby accelerating the nucleation of DRX grains and the formation of Σ3*^n^* twin boundaries. At lower strain rates, there is sufficient time for grain boundary migration, which increases the probability of growth accidents and favors the formation of Σ3*^n^* twin boundaries. Wang et al. [117] conducted a series of hot compression tests at temperatures ranging from 900 °C to 1150 °C and strain rates ranging from 0.01 s^−1^ to 1 s^−1^. The variation in the fraction of Σ3 twin boundaries under different temperatures and strain rates is depicted in Figure 15c. It is shown that during the DRX stage, increasing temperature is conducive to the formation of Σ3 twin boundaries, resulting in an increase in the fraction of Σ3 twin boundaries. When DRX occurs completely, the strain rate exerts a complex influence on the evolution of Σ3 twin boundaries, as also evidenced in Figure 15b. It should be noted that when the temperature is much higher, most interfacial energy is consumed by the grain growth of DRX grains, leading to a reduction in the fraction of Σ3 twin boundaries [61,116,120]. Therefore, the effect of excessive grain growth on the consumption of Σ3*^n^* twin boundaries must be controlled during the thermal deformation process of nickel-based alloys.

**Figure 16:**
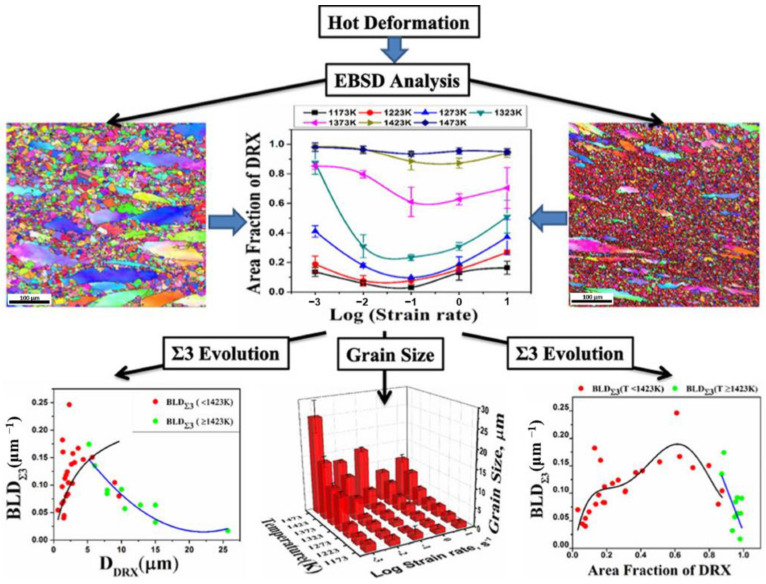
Schematic illustration of analysis processes of DRX and annealing twins in the hot compression process [112].

In addition, some researchers have focused on uncovering the relationship between the evolution of special twin boundaries with deformation parameters and microstructural characteristics during hot compression processes. The analysis process is illustrated in Figure 16. By characterizing the deformed microstructures, the grain size, DRX fraction, the proportion of special twin boundaries involving the twin boundary fraction, and twin density are calculated under different thermal deformation parameters. Subsequently, the relationships between the proportion of special twin boundaries with the deformation parameters, grain size, and DRX fraction can be clarified. Pradhan et al. [112] investigated DRX kinetics and their association with annealing twins during the hot compression process of Alloy 617. The results showed that twin density increases with the increase in the DRX fraction at temperatures below 1423 K, while it decreases with the increasing DRX fraction at temperatures above 1423 K. This suggests that faster grain boundary migration is not favorable for the nucleation of twins during the DRX process of Alloy 617. Shi et al. [66] quantitatively studied the twin boundary characters and derived an analytical model that displays an inversely proportional relationship between Σ3 twin boundary density and DRX grain size during the hot compression process of Ni80A superalloy. Quan et al. [61] conducted the superimposed contour plot maps to uncover the relationships of twin density with grain size and stored energy varying with temperature and strain rate during the thermal deformation process of Nimonic 80A. Recently, Quan et al. [121] proposed a new two-stage deformation method, i.e., prior cold deformation followed by thermal deformation, to improve the GBCD during the thermal deformation process of Nimonic 80A. The results revealed that twin density increases with increasing stored energy and decreasing grain size. Similarly, investigations conducted by Detrois et al. [122], Shi et al. [123], Azarbarmas et al. [124], and others have explored the impact of the deformation parameters on the evolution of Σ3*^n^* twin boundaries in materials such as RR1000 alloy, for GH690, Alloy 718 through the use of hot compression tests. It is evident from these investigations that the deformation parameters have a complex influence on the evolution of Σ3*^n^* twin boundaries. In order to achieve the desired GBCD during the thermal deformation process, the deformation process of alloys can be designed in a favorable thermal processing parameter window that yields a finer grain size and a higher proportion of special twin boundaries.

Table 3 summarizes the optimization results of GBCD during the thermal compression process of nickel-based alloys. These works provide a fundamental understanding of twining-related microstructure evolution and its correlation with the deformation parameters and microstructural characteristics, which assists in realizing microstructure control during the thermal deformation process of nickel-based alloys.

## 4. Development of Twinning-Related Kinetics Models

**Figure 17:**
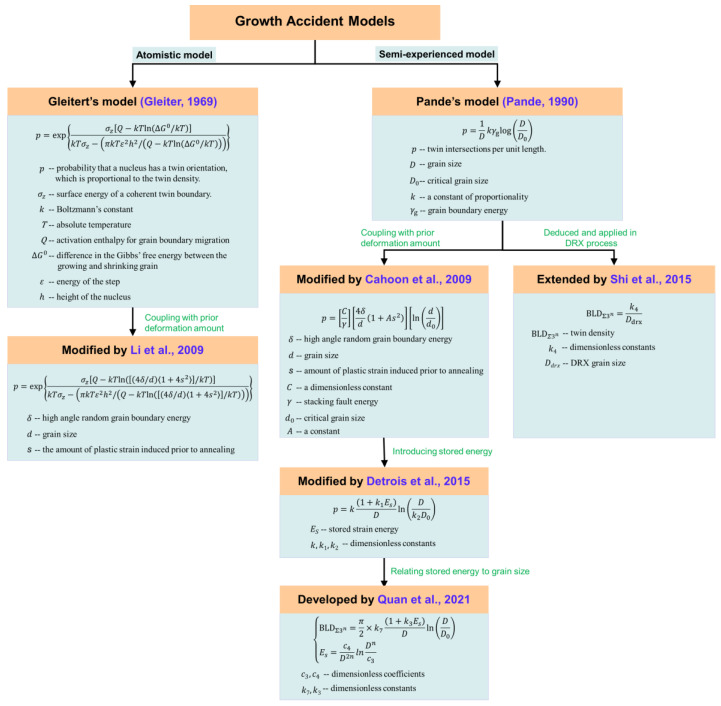
Schematic illustration for the development process of twin density models [21,22,61,62,64,65,66].

Although the influence of the processing parameters on twinning-related microstructures has been extensively studied by numerous researchers, most GBE treatments are still designed through a trial-and-error method based on the experimental data. In order to achieve GBCD optimization effectively, some researchers have focused on developing twinning-related kinetic models that can quantitatively state the evolution of special twin boundaries with processing parameters or microstructure characteristics during twinning-related GBE treatments. This review addresses the development of twinning-related kinetic models. To better illustrate this, Figure 17 schematically depicts the development process of twin density models.

Over the past decades, four main theories have been used to expound the formation of an annealing twin, including the growth accident model [21,22], grain encounter model [23,24,25], grain boundary dissociation [26,27,28], and the twin nucleation model [29,30]. Among these, the growth accident model, which posits that the annealing twin boundary is generated at grain boundary migration owing to a stacking error occurring under energetically favorable conditions, has gained widespread recognition among researchers. Gleiter [21] elaborated the formation mechanism of annealing twins in an atomistic view of the twin interface based on the growth accident model and proposed a complicated expression for twin formation probability to quantify annealing twin formation. It must be highlighted that in Gleiter’s model, many material parameters are difficult to determine, which mitigates the direct applicability of this model. Thereupon, Pande [22] reviewed the growth accident model and proposed a semi-experienced function for predicting twin density in grain growth. According to Pande’s model, grain size and grain boundary energy are the key factors affecting final twin density.

With the development of twinning-related GBE, the manipulation of twin boundary density is no longer confined to the annealing process alone. It has been discovered that the combination of prior deformation with the annealing process, known as TMP treatment, is regarded as an attractive approach to effectively improving the fraction of special twin boundaries efficiently [3,37,38,80,101,102]. Considering the influence of prior deformation on annealing twin density, Li et al. [62] modified Gleiter’s model by incorporating the prior deformation amount into Gibbs’ free energy. Similarly, Cahoon et al. [64] introduced the prior amount of deformation into Gibbs’ free energy term to modify Pande’s model. Meanwhile, they substituted the dimensional but not the physical meaning coefficient in Pande’s model with the stacking fault energy of materials. The final twin density model is as a function of grain size, stacking fault energy, and the prior amount of deformation. Detrois et al. [65] deemed that the works of Li et al. and Cahoon et al. had limitations because they only accounted for the effect of prior strain in their modified terms of Gibbs’ free energy. For a prior thermal deformation process, the formation of an annealing twin is significantly influenced by three parameters, including temperature, strain rate, and strain. In this regard, Detrois et al. modified the Gibbs’ free energy terms using stored strain energy that is closely associated with the three parameters, where the stored strain energy can be calculated from EBSD maps. The above works are dedicated to the development of twin density models in static processes, such as recrystallization or grain growth.

For some materials, such as nickel-based alloys, their components are typically manufactured usig a thermal forming process to achieve excellent mechanical properties. It is necessary to describe the evolution of twin boundaries during dynamic thermal deformation processes. Based on the fundamental assumption in Pande’s model, Shi et al. [66] derived an inversely proportional relationship between twin density and DRX grain size to quantitatively describe the evolution of twin boundary density during a thermal–plastic deformation process of a nickel-based alloy. Based on the work of Detrois et al., Quan et al. [61] developed an improved twin density model related to stored energy and grain size to predict the twin density evolution during the thermal deformation process of Ni80A superalloy. In their work, the stored energy was further considered to be a function of average grain size, with the aim of relating the twin density to the underlying thermal deformation variables. It is important to mention that there is no universal model that can describe the evolution of twin density in all cases, and thus it is imperative to further develop the twin density model.

## 5. Application of Twinning-Related Grain Boundary Engineering

Since the conception of GBE was proposed, plenty of studies have been conducted to optimize GBCD to improve the grain-boundary-related properties of FCC materials, which provides an effective approach to enhancing the performance of components. This section highlights some case studies related to improving the mechanical properties and corrosion resistance of FCC materials through twinning-related GBE. Furthermore, a thorough investigation of the underlying mechanisms responsible for strengthening the properties is provided.

### 5.1. Improvement of Mechanical Properties

**Figure 18:**
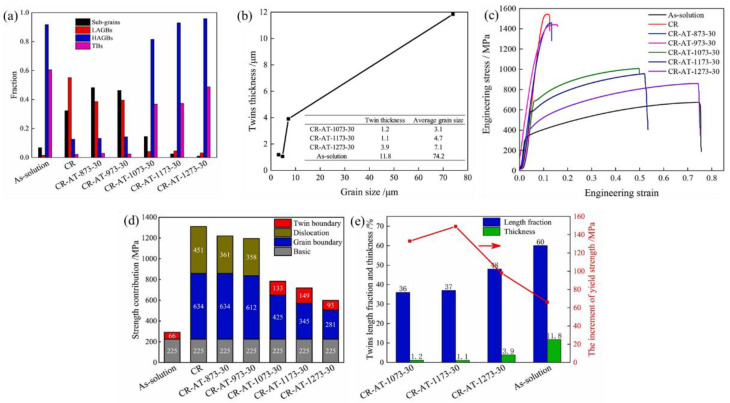
GBCD results and tensile mechanical properties of 625 alloy [6]. (**a**) Fractions of twin boundaries; (**b**) twining thinness; (**c**) tensile stress–strain curves; (**d**) strength contribution; (**e**) relationships between yield strength increment, fraction of annealing twin boundaries, and twin thickness.

This section presents a comprehensive overview of the impact of the twinning-related GBE microstructures on the mechanical properties of FCC materials with low or medium SFE, such as strength, plasticity, fatigue, and creep behaviors. The results of Detroits et al. [122] showed that the compressive strength increases as the fraction of the Σ3 twin boundary increases for the specimens of RR1000 nickel-based superalloy that underwent hot compression tests under temperatures of 1020~1100 °C. Sinha et al. [128] and Kumar et al. [129], respectively, investigated the twinning-related microstructures and tensile mechanical properties of 18Cr-9Ni and 304L austenitic stainless steels under different TMP routes. The results suggested that the specimen after TMP treatment exhibits greater ductility owing to the special twin boundaries. Gao et al. [130] demonstrated that the strength and ductility of Alloy 625 could be substantially enhanced through TMP treatment. After hot/cold deformation and subsequent heat treatment, the yield strength and elongation of specimens are greatly increased from 247 MPa and 41% to 366 MPa and 60%, 361 MPa and 52.88%. Wang et al. [6] realized the strength–ductility synergy of Alloy 625 through cold rolling and subsequent annealing treatment. They suggested that the improved strength and ductility are attributed to grain boundary strengthening and twin boundary strengthening mechanisms. The strengthening effect of annealing twins depends on twinning thickness rather than the fraction of twin boundaries. Figure 18a–e depict the GBCD results and tensile mechanical properties of Alloy 625 subjected to various TMP routes. The specimens after TMP treatment exhibit excellent ductility and high strength. Specifically, for the specimen that underwent cold rolling and followed by annealing at 1073 K for 30 min, its yield strength increased to 676 MPa, which is approximately 2.3 times higher than that of the as-solution specimen. Additionally, the elongation is maintained as high at 50%. Figure 18d,e demonstrate the strength contribution and the relationships between yield strength increment, the fraction of annealing twin boundaries, and twin thickness. It is revealed that with the increase in annealing temperature, the strength contribution of the twin boundary decreases with the increasing fraction of twin boundaries, while the yield strength increment increases with decreasing twin thickness.

**Figure 19:**
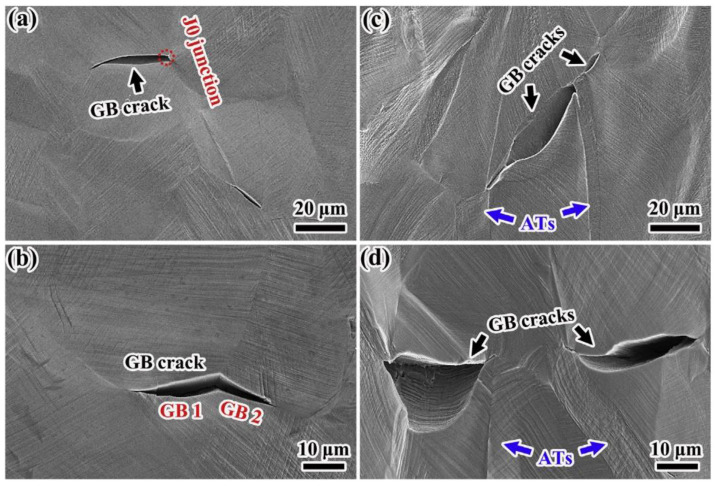
SEM morphologies highlighting the deformation and cracking features on the lateral surface of BM (**a**,**b**) and GBE (**c**,**d**) specimens tensioned to rupture [7].

Guan et al. [7] investigated the influence of GBCD optimization on the tensile properties of FCC Cu-16at.%Al alloy, and it was found that the GBE specimen exhibited superior plasticity compared to the base material (BM) specimen. Figure 19 shows the crack morphologies observed on the lateral surface of BM and GBE specimens tensioned to rupture. The results indicated that the cracks in the BM and GBE specimens germinate at random HAGBs. In the case of the BM specimen, these cracks propagate along random HAGBs toward the J0 junction (Figure 19a) or other random HAGBs (Figure 19b), while the low-ΣCSL boundaries formed in the GBE specimen hinder inter-crack penetration (Figure 19c,d), thereby retarding crack propagation. Guan et al. [131] also conducted tension–tension fatigue tests on non-GBE and GBE specimens of this alloy. They suggested that intergranular fatigue cracking can be effectively suppressed through the application of an appropriate GBE treatment, thereby improving the low-cycle fatigue life of FCC materials.

**Figure 20:**
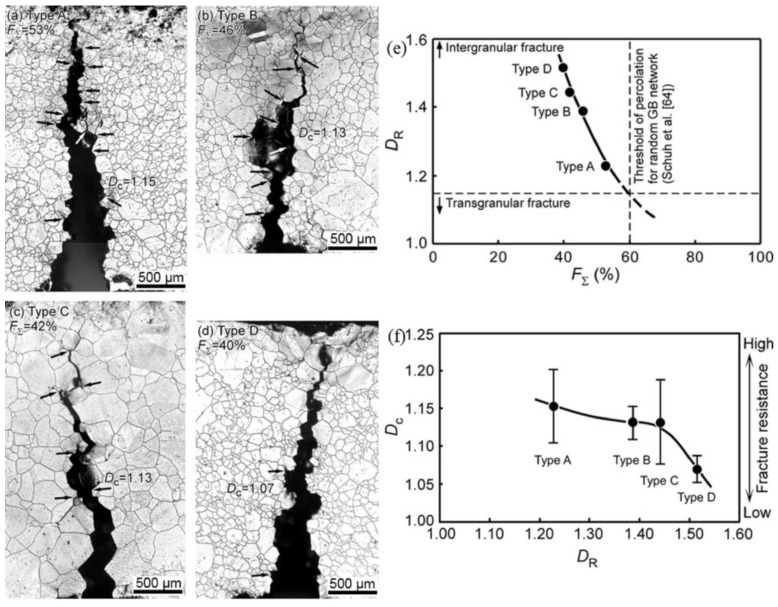
Crack path micrographs for four different types specimens (**a**–**d**), the relationships between *D*_R_ and *F*_Σ_ (**e**), between *D*_c_ and *D*_R_ (**f**) [13]. The arrows in the micrographs indicate the branched cracks.

Thaveeprungsriporn et al. [80] analyzed the impact of CSL boundaries on the creep deformation of Ni-16Cr-9Fe at 360 °C. The results suggested that when the fraction of CSL boundaries is increased by about twofold, the steady-state creep rates can be decreased 8~26 times for the coarse-grain specimen (330 μm) and 40~66 times for the finer-grain specimen (35 μm). Kobayashi et al. [13] evaluated the effect of twinning-related microstructures on the fracture resistance of polycrystalline nickel and revealed that a higher fraction of low-ΣCSL boundaries and a lower fractal dimension in the random boundary network connectivity result in greater fracture resistance. Figure 20a–d display the micrographs of the crack path in four different types of specimens. It is evident that the crack path is frequently deflected and branched in the type A fractured specimen, while crack branching hardly occurred in type D fractured specimens. Figure 20e,f demonstrate the relationships between the fractal dimension of random boundary network connectivity (*D*_R_) with the fraction of low-ΣCSL boundaries (*F*_Σ_) and the fractal dimension of the crack path (*D*_c_). It can be seen in Figure 20e,f that *D*_R_ decreases with the increasing fraction of low-ΣCSL boundaries, *D*_c_ decreases with increasing *D*_R_, which indicates that lower *D*_R_ with more frequent branching of cracks may result in higher *D*_c_. Therefore, the specimen with higher *F*_Σ_ and lower *D*_R_ shows higher fracture resistance by restricting more frequent branching and the deflection of the propagating crack path along random HAGBs from the main crack.

### 5.2. Improvement of Corrosion Resistance

**Figure 21:**
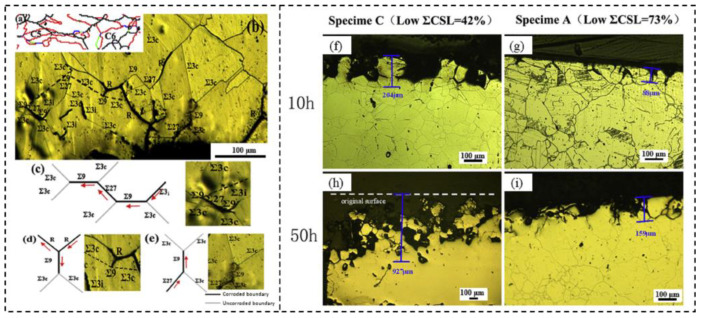
The propagation path of IGC in the cross-section of specimen A (**a**–**e**) and micrographs of specimen C and specimen A subjected to 10 h and 50 h corrosion (**f**–**i**) [79].

The corrosion resistance of materials can be usually enhanced by modifying alloy chemistry or applying protective coatings [132,133,134]. Corrosion occurs when alloying element are depleted from the base material or reactive elements penetrate into it from the environment. These phenomena involve diffusion through random grain boundaries, which offer a relatively easy pathway due to their open structure. The existence of twin boundaries can significantly improve the corrosion resistance of materials. Considerable efforts have been devoted to improving the intergranular corrosion (IGC) and intergranular stress corrosion cracking (IGSCC) of FCC materials through GBCD optimization. Ma et al. [5] for 625 alloy, Shi et al. [71] for 18Cr-18Mn-0.63N austenitic stainless steel, Shimada et al. [3] and Feng et al. [90] for 304 stainless steel, Pradhan et al. [40] for 304L austenitic stainless steel, Yuan et al. [82] for high purity copper, optimized the GBCD through TMP treatments, and revealed that the improvement of IGC resistance results from the increased fraction low-ΣCSL boundaries or the disrupted connectivity of random boundary network. It is noted that not all types of low-ΣCSL boundaries exhibit strong inhibition of IGC cracks. Shi et al. [94] suggested that only Σ3 twin boundaries play an effective role in the IGC resistance of Fe-20Cr-19Mn-2Mo-0.82N austenitic stainless steel, while random HAGBs and Σ9, Σ11, and Σ27 twin boundaries do not withstand IGC under the experimental conditions. Hu et al. [79] improved the IGC resistance of 304 stainless steel by controlling GBCD. They reported that only coherent Σ3 twin boundaries exhibit resistance to IGC. The microstructures characterized as large grain clusters with a high proportion of Σ3*^n^* twin boundaries and interconnected Σ3*^n^* triple junctions contribute to enhancing the IGC resistance. Figure 21a–e illustrates the propagation path of IGC in the cross-section of specimen A of 304 stainless steel. The random HAGBs are clearly found to be corroded heavily, while almost all coherent Σ3 twin boundaries (Σ3c) within grain clusters exhibit no evidence of corrosion (Figure 21a,b). Moreover, the intergranular attacks also propagate through Σ9 and Σ27 twin boundaries, but they are arrested by two coherent Σ3 twin boundaries in a triple junction of Σ3-Σ3-Σ9, as shown in Figure 21c–e. The micrographs of specimen C and specimen A subjected to 10 h and 50 h corrosion were presented in Figure 21f–i. It was revealed that the penetration depth of intergranular attacks increases with corrosion time increasing. Due to the large grain clusters and the high proportion of low-ΣCSL boundaries, the penetration depth for specimen A is relatively lower, indicating superior resistance to IGC.

**Figure 22:**
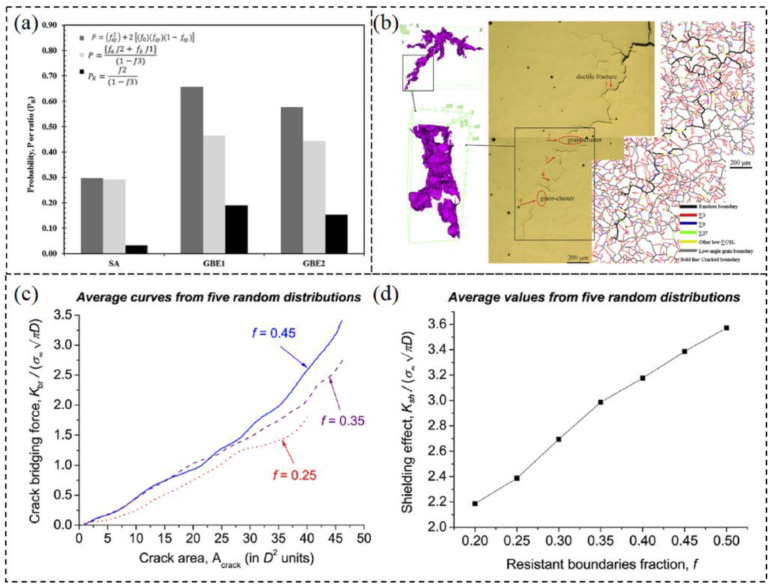
(**a**) The crack arrest probability for Alloy 600 under different GBE treatments [93]; (**b**) large crack bridges in the microstructure of 316 stainless steel [136]; (**c**) evolution of crack-bridging force with crack area and resistant boundaries fraction [137]; (**d**) relationship between the shielding effect of crack bridges and resistant boundaries fraction [137].

Percolation theory and crack-bridging ligament are commonly employed by researchers to elucidate the impact of twinning-related microstructures on the IGSCC resistance of FCC materials. When the proportion of special twin boundaries reaches a permeation threshold, the connectivity of the random boundary network can be adequately interrupted, resulting in large grain clusters. The triple junctions formed within these grain clusters contribute to improving the resistance of materials to intergranular cracks. The crack arrest probability is typically calculated using three percolation models proposed by Kumar et al. [95], Palumbo et al. [96], and Marrow et al. [97]. Telang et al. [93] investigated the influence of iterative TMP treatment on the corrosion resistance and stress corrosion cracking (SCC) of Alloy 600. The results indicated that in the microstructures after TMP treatment, the disrupted connectivity of the random boundary network and the increased fraction of low-ΣCSL boundaries hinder the propensity of carbide precipitation and Cr depletion. Based on the percolate models, the probability for crack arrest at low-ΣCSL boundaries and triple junctions (1-CSL and 2-CSL types) was calculated, as shown in Figure 22a. The results indicated an increase in crack arrest probability, suggesting improved resistance to corrosion and SCC after TMP treatment. Shi et al. [135] optimized GBCD to enhance the IGSCC resistance of Fe-18Cr-17Mn-2Mo-0.85N high-nitrogen nickel-free austenitic stainless steel. It was suggested that high proportions of low-ΣCSL boundaries and special triple junctions (2-CSL and 3-CSL types) contribute to enhancing the IGSCC resistance of high-nitrogen steel by hindering the precipitation of intergranular nitrides along grain boundaries and preventing the intergranular propagation of cracks. Liu et al. [136] revealed that large grain clusters have a significant effect on the enhancement of the IGSCC of 316 stainless steel. The main intergranular cracks propagated in a zigzag along the outer boundaries of large grain clusters, as the inner Σ3*^n^* twin boundaries have lower susceptibility to SCC than random HAGBs. These large grain clusters possess complex morphologies and get tangled, making them difficult to separate during IGSCC. This results in many large crack bridges remaining on the crack surface, as exhibited in Figure 22b. Jivkov et al. [137] simulated the IGSCC propagation and crack coalescence using a three-dimensional mechanical model. Figure 22c,d exhibits the influence of resistant boundaries fraction on the crack-bridging force and shielding effect of crack bridges. The results indicated that a higher fraction of resistant boundaries could provide greater crack-bridging force. Increasing the fraction of resistant boundaries is conducive to increasing the degree of crack tip shielding developed, thus improving the resistance to IGSCC.

## 6. Conclusions and Future Prospects

GBE is a highly effective approach for improving grain-boundary-related properties through the optimization of GBCD. This article provides a detailed review concerning controlling GBCD during the twinning-related GBE of FCC materials. The following are some critical conclusions that can be drawn from this review.

(1) This review introduces the fundamentals of twinning-related GBE, encompassing the definition of special boundaries, the morphology of the annealing twin, the microscopic mechanisms required to optimize GBCD, and the optimization objectives of GBCD. The nature of optimizing GBCD in the twinning-related GBE of FCC materials is to improve the proportion of special twin boundaries and sufficiently interrupt the connectivity of the random boundary network.
(2) The TMP treatment is the primary processing route used to achieve GBE. The initial microstructures, amount of deformation, annealing temperature and time, and processing pass are critical processing parameters that significantly impact the formation of special twin boundaries. Determining the optimal combination of TMP parameters still relies on trial-and-error or experience methods, and the control of GBCD through adjusting TMP parameters remains a challenging task that requires further attention.
(3) This review addresses the influence of the deformation parameters on the evolution of special twin boundaries during the thermal deformation process of nickel-based alloys. Microstructural evolution mechanisms are extremely intricate, and the proportion of special twin boundaries exhibits a non-linear relationship with the deformation parameters and microstructural characteristics. To achieve the desired GBCD, the thermal deformation process of alloys can be designed within a favorable processing parameter window that yields a finer grain size and a higher proportion of special twin boundaries.
(4) This review presents the development process of twinning-related kinetics models associated with processing parameters or microstructure characteristics. However, there is no universal model that can describe the evolution of twin density in all cases. The twinning-related kinetics model will be further developed in future studies.
(5) This review provides some applications of twinning-related GBE in terms of enhancing the mechanical properties and corrosion resistance of FCC materials. The results suggest that the microstructures with a high proportion of special twin boundaries and the disrupted connectivity of the random boundary network contribute significantly to enhancing the mechanical properties and corrosion resistance of the materials.

## Figures and Tables

**Table 1:** List of low-ΣCSL boundaries (Σ ≤ 29) and their corresponding rotation axes <uvw> and angles *θ* in cubic crystal system [54,78].

Σ	*θ*	<uvw>	Σ	*θ*	<uvw>	Σ	*θ*	<uvw>
1	0°	<111>	13b	27.8°	<111>	21b	44.4°	<211>
3	60°	<111>	15	48.2°	<210>	23	40.5°	<311>
5	36.9°	<100>	17a	28.1°	<100>	25a	16.3°	<100>
7	38.2°	<111>	17b	61.9°	<221>	25b	51.7°	<331>
9	38.9°	<110>	19a	26.5°	<311>	27a	31.6°	<110>
11	50.5°	<110>	19b	46.8°	<100>	27b	35.4°	<210>
13a	22.6°	<100>	21a	21.8°	<331>	29	46.3°	<100>

where a and b are the same low-ΣCSL boundaries resulting from the rotation of different angles *θ* around different axis <uvw>.

**Table 2:** Optimization results of GBCD during the TMP treatment of some FCC materials.

Material		Initial State or Pre-Treatment	Optimal TMP Route				GBCD Results			Grain Size /μm	Ref.
			Reduction /%	Temperature /°C	Time /min	Processing Pass/n	Fraction of Low-ΣCSL/%				
							Σ3	Σ9 + 27	Σ ≤ 29		
Nickel-based alloy	690	1100 °C/15 min + WQ	5	1100	5	1	60.6	9.2	72.5	-	[102]
	690	1100 °C/15 min + WQ + 750 °C/15 h	5	1100	5	1	61.3	9.4	72.7	-	[87]
	Ni-200	Strips	5	900	10	1	50.2	11.1	65.0	67	[103]
	Ni	As-cast	60	950~1150	1~15	1	46.7	5.5	66.2	25	[107]
	625	1150 °C/1 h + WQ	5	1150	5	1	30.3	3.2	35.8	41.5	[5]
	600	Bar	20	1000	15	7	~34	-	~65	-	[95]
	-	1000 °C/1 h	65	1020	180	1	40.3	-	41.5	31.3	[42]
	Ni-200	Sheet	6	900	10	1	-	-	74.7	60	[37]
	Pure nickel	Sheet	6	700	2880	1	59.8	13.5	76.1	46	[43]
	600 H	1130 °C + WQ	7.5	1100	60	1	~64	~8	72	74	[39]
	825	Solution-annealed	5	1050	10	1	-	-	77.6	12.47	[58]
	925	1100 °C/2 h + WQ	7	1075	10	1	~56	~5	-	~63	[108]
	617	1175 °C + WQ	15	1100	30	1	~67	-	-	~74	[109]
Austenitic stainless steel	316	1100 °C/0.5 h	3	967	4320	1	-	-	86	-	[101]
	304	1100 °C/15 min + WQ	5	1100	5	1	59.2	8.7	73.1	33	[79]
	304	1050 °C/30 min	5	927	4320	1	-	-	86.5	-	[3]
	304L	Hot rolling + 1050 °C/1 h + WQ	5	1000	60	3	72	9	-	30	[40]
	304 L	1070 °C/1 h + WQ	90	950	30	1	37	-	41	~20	[41]
	316H	1060 °C/1 h + WQ	5	1010	120	1	67.4	8.9	76.3	-	[35]
	D9	Solution-annealed	10	1000	30	2	62	~11	73	~50	[45]
	Fe-Cr-Mn-Mo-N	1050 °C/1 h + WQ	5	1150	4320	1	74	5.2	79.4	25	[94]
Pb-alloy	Pb–Ca–Sn	Strip cast + 90% rolling	30	270	10	2	63.9	25.8	96.1	60	[105]
	Pb–Ca–Sn–Al	90% cold rolling + 270 °C/15 min	30	270	10	3	59.9	11	80.0	-	[32]
	Pb–Ca	90% cold rolling + 270 °C/10 min	30	270	10	3	53	~2	~57	-	[46]
Brass	Cu–Zn	25% hot rolling + 800 °C/1 h	20	680	20	2	73	5	79	42	[106]
Ofe-Cu		Plate	6	270 °C/14 h + 375 °C/7 h		1	65	15	85	-	[110]
		Bar	67	560	10	4	45	-	68	30	[27]
		750 °C/15 min	5	700	15	1	-	-	~83.5	~77	[59]

**Table 3:** Optimization results of GBCD under different thermal deformation parameters for nickel-based alloys.

Nickel-Based Alloy	Initial State or Pre-Treatment	Initial Grain Size/μm	Hot Compression Parameters			Maximum Value of Low-ΣCSL			Ref.
			Temperature /°C	Strain Rate /s^−1^	True Strain or Reduction	Fraction of Low-ΣCSL/%			
						Σ3	Σ9	Σ27	
Ni80A	1065 °C × 8 h	170	1120–1180	0.1, 1	1.1	48	~2.6	-	[66]
-	Ingot casting, 1150 °C × 30 min	38.93	900–1150	0.01–1	50%	35	-	-	[117]
617B	1210 °C × 48 h	94	1120–1210	0.001	0.16~0.84	~18	~1.4	~0.49	[116]
617	-	-	900–1200	0.001–10	50%	~53	-	-	[112]
718	1025 °C × 1 h	45	950–1100	0.001–1	0.05~0.7	-	-	-	[124]
718	Wrought billet, 1100 °C × 30 min	176	950–1120	10	0.7	-	-	-	[125]
Monel 400	-	220	750–1150	0.01	0.7	-	-	-	[126]
800H	Hot forged	-	850–1100	0.01–10	0.7	~40	~2.1	~0.9	[118]
GH690	Hot forged	130	1000–1200	0.001–10	50%	~28	~0.9	~0.2	[123]
-	Wrought billet, 1100 °C × 30 min	95	950–1100	0.01–1	0.357	~21	-	-	[127]
-	Forging bar, 1100 °C × 30 min	96.5	1100	0.001–10	0.7	~30	~2.4	~0.8	[119]
-	Forging bar, 1100 °C × 0.5 h	91.6	960–1160	0.001–1	50%	~30	~2.4	~0.8	[120]
RR1000	As-consolidated billet, 1110 °C × 4 h	3.2	1020–1100	0.001–0.05	0.5	33	-	-	[122]

## Data Availability

Not applicable.
